# Anthracyclines suppress pheochromocytoma cell characteristics, including metastasis, through inhibition of the hypoxia signaling pathway

**DOI:** 10.18632/oncotarget.16224

**Published:** 2017-03-15

**Authors:** Ying Pang, Chunzhang Yang, Jan Schovanek, Herui Wang, Petra Bullova, Veronika Caisova, Garima Gupta, Katherine I. Wolf, Gregg L. Semenza, Zhengping Zhuang, Karel Pacak

**Affiliations:** ^1^ Section on Medical Neuroendocrinology, Eunice Kennedy Shriver National Institute of Child Health and Human Development, National Institutes of Health, Bethesda, Maryland, USA; ^2^ Neuro-Oncology Branch, Center for Cancer Research, National Cancer Institute, Bethesda, Maryland, USA; ^3^ Department of Internal Medicine III-Nephrology, Rheumatology, and Endocrinology, Faculty of Medicine and Dentistry, Palacky University, Olomouc, Czech Republic; ^4^ Surgical Neurology Branch, National Institute of Neurological Disorders and Stroke, National Institutes of Health, Bethesda, Maryland, USA; ^5^ Department of Molecular Medicine, Institute of Virology, Biomedical Research Center, Slovak Academy of Sciences, Bratislava, Slovak Republic; ^6^ McKusick-Nathans Institute of Genetic Medicine and Institute for Cell Engineering, Johns Hopkins University School of Medicine, Baltimore, Maryland, USA

**Keywords:** anthracyclines, paraganglioma, pheochromocytoma, metastatic, hypoxia-inducible factors

## Abstract

Pheochromocytomas (PHEOs) and paragangliomas (PGLs) are rare, neuroendocrine tumors derived from adrenal or extra-adrenal chromaffin cells, respectively. Metastases are discovered in 3-36% of patients at the time of diagnosis. Currently, only suboptimal treatment options exist. Therefore, new therapeutic compounds targeting metastatic PHEOs/PGLs are urgently needed. Here, we investigated if anthracyclines were able to suppress the progression of metastatic PHEO. We explored their effects on experimental mouse PHEO tumor cells using *in vitro* and *in vivo* models, and demonstrated that anthracyclines, particularly idarubicin (IDA), suppressed hypoxia signaling by preventing the binding of hypoxia-inducible factor 1 and 2 (HIF-1 and HIF-2) to the hypoxia response element (HRE) sites on DNA. This resulted in reduced transcriptional activation of HIF target genes, including erythropoietin (*EPO*), phosphoglycerate kinase 1 (*PGK1*), endothelin 1 (*EDN1*), glucose transporter 1 (*GLUT1*), lactate dehydrogenase A (*LDHA*), and vascular endothelial growth factor (*VEGFA*), which consequently inhibited the growth of metastatic PHEO. Additionally, IDA downregulated hypoxia signaling by interfering with the transcriptional activation of *HIF1A* and *HIF2A*. Furthermore, our animal model demonstrated the dose-dependent suppressive effect of IDA on metastatic PHEO growth *in vivo*. Our results indicate that anthracyclines are prospective candidates for inclusion in metastatic PHEO/PGL therapy, especially in patients with gene mutations involved in the hypoxia signaling pathway.

## INTRODUCTION

Pheochromocytomas (PHEOs) and paragangliomas (PGLs) are rare neuroendocrine tumors located in the adrenal medulla and extra-adrenal paraganglia, respectively. Although most PHEOs/PGLs are benign, approximately 10% of PHEOs and 15-35% of PGLs are metastatic, irrespective of their non-hereditary or hereditary status [1, 2]. Established in the 1980s, the current conventional chemotherapy regimen combines cyclophosphamide, vincristine, and dacarbazine (CVD) [3]. Despite its popularity, the long-term application of CVD is often limited due to a short remission period and toxicity limitations [4].

Intratumoral hypoxia plays a major role in cancer recurrence, spread, and resistance to radiotherapy and chemotherapy [5-7]. A major consequence of reduced O_2_ availability in tumors is the activation of hypoxia-inducible factors (HIFs) [8-11]. HIF-1α and HIF-2α are stabilized and can each dimerize with HIF-1β and bind to hypoxia response elements (HREs) to transactivate various target genes involved in angiogenesis, immune system modulation, iron metabolism, erythropoiesis, glucose metabolism, and other physiological processes [12]. Although HIF-1α dysregulation has been found in a wide variety of cancers [12], several studies indicate that HIF-2α upregulation is more commonly associated with an aggressive phenotype and poor prognosis in neural crest cell tumors, including PHEO/PGL and neuroblastoma [13-18]. Therefore, HIF-1α and HIF-2α inhibitors should be considered for therapeutic management of these tumors.

Anthracyclines are a well-known class of chemotherapy drugs, mainly consisting of daunorubicin (DAU), doxorubicin (DOX), epirubicin (EPI), and idarubicin (4-demethoxydaunorubicin, IDA), and when administered to mice on a low-dose, daily (metronomic) schedule, have been shown to suppress tumor growth and angiogenesis by blocking the HIF signaling pathway [19, 20]. Over the past 50 years, anthracyclines have been widely used in the treatment of multiple cancer types, including leukemias, breast, bladder, and thyroid carcinoma [21, 22]. In 2003, successful results were reported in a patient with rapidly progressive, metastatic PHEO treated with a combination of CVD and anthracyclines [23]. However, the use of anthracyclines in the treatment of metastatic PHEO/PGL has not been further evaluated in any published or experimental studies.

In the present study, we assessed the effects of three anthracyclines (EPI, DOX, and IDA) on the proliferation and migration of an experimental mouse PHEO cell line. Our report investigated the mechanisms by which anthracyclines suppress tumor cell growth and the HIF signaling pathway using an *in vitro* cell model. Furthermore, for the first time, the effect of anthracyclines was evaluated in nude mice bearing a metastatic PHEO allograft, in order to explore their potential use in the treatment of metastatic PHEO/PGL.

## RESULTS

### Anthracyclines suppress cell growth and migration

**Figure 1:**
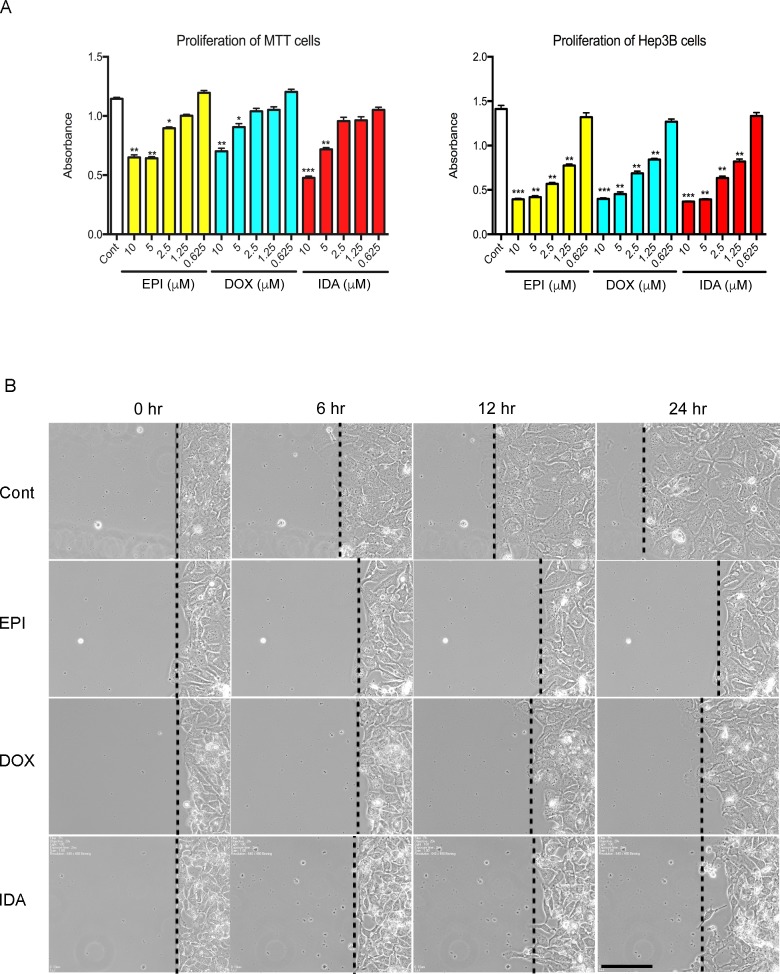
Effects of anthracyclines on cell proliferation and cell migration **A**. MTT or Hep3B cells were seeded in a 96-well plate. Cell proliferation was assessed under treatment of EPI, DOX, or IDA at different concentrations (10, 5, 2.5, 1.25, or 0.625 μM). Control versus anthracycline treatment, *P < 0.05; **P < 0.01; ***P < 0.001. **B**. Cell migration on a confluent cell monolayer was observed for 24 hours under treatment of EPI, DOX, or IDA at a concentration of 0.625 μM. DMSO was used as the control group. Bar = 100 μm.

To assess the impact of anthracyclines on PHEO cells *in vitro*, the proliferation of Mouse Tumor Tissue (MTT) cells, a cell line that was generated from a mouse PHEO, was analyzed in the presence of EPI, DOX, or IDA. Anthracyclines inhibited MTT proliferation in a dose-dependent manner. Compared to the control group, higher concentrations of EPI, DOX, and IDA (5 μM and 10 μM) led to statistically significant reductions in cell number (Figure 1A). By contrast, low concentrations (0.625 μM) of EPI, DOX, or IDA did not cause obvious cytotoxicity during the 16-hour treatment period. The half maximal inhibitory concentration (IC_50_) of EPI, DOX, and IDA was 7.0 μM, 11.6 μM, and 6.0 μM, respectively. Additionally, anthracyclines showed significant suppressive effects on Hep3B cell proliferation (Figure 1A).

A concentration of 0.625 μM, which did not cause increased cell death, was chosen to perform a more detailed mechanistic study. To test whether anthracyclines inhibit cell motility, a scratch assay was used over a 24-hour period. Compared to the control group, all three anthracycline compounds exhibited an inhibitory effect on the motility of Hep3B cells (Figure 1B).

### IDA interferes with HIF-1α and HIF-2α mRNA expression

**Figure 2:**
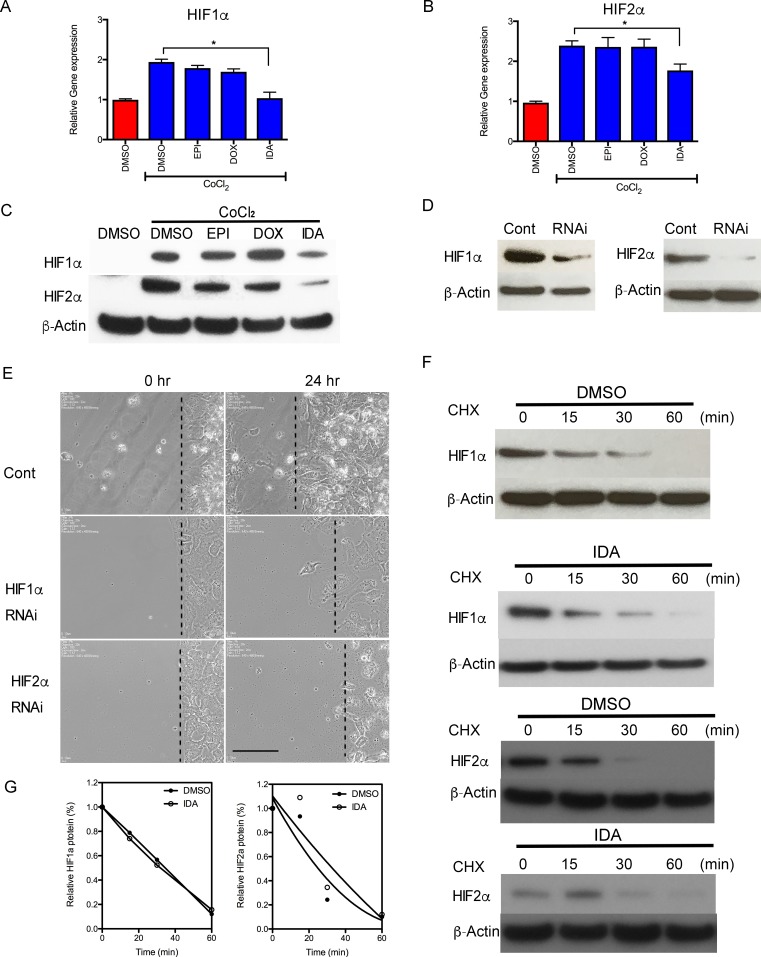
Anthracyclines interfere with the transcription of HIF-1α and HIF-2α **A**.-**B**. Hep3B cells were exposed to CoCl_2_ in the presence of DMSO (Control) or 0.625 μM EPI, DOX, or IDA for 16 hours. HIF-1α and HIF-2α mRNA levels were measured by RT-qPCR. Anthracycline treatment under CoCl_2_ was compared with CoCl_2_ treatment alone. *P < 0.05. **C**. HIF-1α and HIF-2α protein levels, in cells treated with CoCl_2_ in the presence of DMSO or EPI, DOX, or IDA, were determined by immunoblot assays. β-Actin was used as the internal control. **D**. Knock-down effects of HIF-1α and HIF-2α siRNA were verified by western blot. β-Actin was performed as the internal control. **E**. siHIF-1α and siHIF-2α were used to knock down gene expression and their inhibitory effects on cell migration were observed. Bar = 100 μm. **F**. Cells were incubated with or without CHX (100 μM) and IDA (0.625 μM) for the indicated incubation times. Protein level changes of HIF-1α and HIF-2α were measured by western blot. **G.** Quantification and half-life of HIF-1α and HIF-2α under IDA treatment with DMSO was used as the control vehicle.

Since we needed to study the effects of anthracyclines on HIF signaling pathways and HIF target genes under hypoxic conditions, in the following *in vitro* studies, Hep3B cells were used instead of MTT cells due to the suboptimal sensitivity of MTT cells to hypoxia. To determine the activity of HIF-1α and HIF-2α under anthracycline treatment, cellular protein levels of HIF-1α and HIF-2α were analyzed in the presence of CoCl_2_, a chemical inducer of HIF activity. After treatment with CoCl_2_ for 16 hours, HIF-1α and HIF-2α mRNA and protein levels were significantly increased compared to the control group. IDA blocked the increase in HIF-1α and HIF-2α mRNA and protein levels, whereas EPI and DOX did not exert obvious inhibitory effects (Figure 2A, 2B, and 2C).

To investigate whether anthracyclines blocked cell migration via inhibition of HIF activity, the effect of drug treatment was compared with the effect of treatment with siRNA targeting HIF-1α and HIF-2α in Hep3B cells without CoCl_2_ treatment. Figure 2D shows the efficiency of knocking down HIF-1α or HIF-2α by siRNA. Consistent with anthracycline treatment, migration of the siHIF-1α or siHIF-2α transfected cells decreased compared to the control siRNA group (Figure 2E).

We then investigated the effect of IDA on the degradation of HIF-1α and HIF-2α. Co-treatment with cycloheximide to block *de novo* protein synthesis did not decrease the half-life of either HIF-1α or HIF-2α compared to the control (Figure 2F and 2G). Taken together, these results suggest that IDA down-regulated HIF-1α and HIF-2α expression in part by reducing HIF-1α and HIF-2α mRNA levels, without affecting protein stability.

### Anthracyclines suppress transcriptional activity by blocking binding of HIFs to DNA

**Figure 3:**
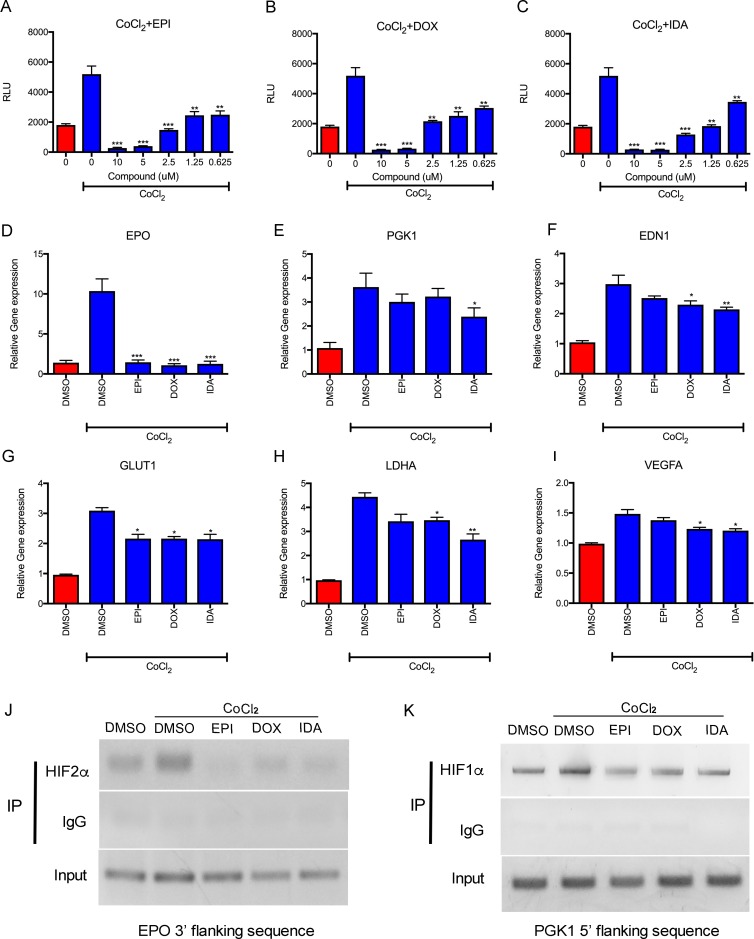
Anthracyclines suppress HIF transcriptional activity **A**.-**C**. Luciferase reporter assay showed that EPI, DOX, and IDA blocked the HRE luciferase activity of HIFs in a dose-dependent manner. Anthracycline treatment under CoCl_2_ was compared with CoCl_2_ treatment alone. *P < 0.05; **P < 0.01; ***P < 0.001. **D**.-**I**. The effects of EPI, DOX, and IDA on mRNA levels of HIF-1α or HIF-2α target genes were measured by qRT-PCR and reduced under CoCl_2_-induced hypoxia conditions, showing obvious inhibition on *EPO, PGK1, EDN1, GLUT1, LDHA,* and *VEGFA*. Anthracycline treatment under CoCl_2_ was compared with CoCl_2_ treatment alone. *P < 0.05; **P < 0.01; ***P < 0.001. **J**.-**K**. Analysis of HIF DNA binding was assessed by ChIP assays. Input DNA was isolated from whole cell lysates. The remaining lysate was divided and incubated with HIF-1α, HIF-2α or IgG antibodies for IP. The 3′ flanking region of EPO and the 5′ flacking region of *PGK1* were amplified from the immunoprecipitates. One percent agarose gels were run to analyze PCR products.

The effects of anthracyclines on HIF transcriptional activity were evaluated via the luciferase assay using the Hep3B HRE luciferase cell line, which carries the HRE reporter. EPI, DOX, and IDA down-regulated CoCl_2_-induced HRE luciferase activity in a dose-dependent manner. Additionally, a concentration as low as 0.625 μM showed significant suppressive effects on transcription (Figure 3A, 3B, and 3C). The expression of endogenous HIF target genes (*EPO, PGK1, EDN1, GLUT1, LDHA, and VEGFA*) was also analyzed. In accordance with HRE luciferase activity, anthracyclines significantly reduced the expression of these genes (Figure 3D-3I). *EPO* expression was reduced to basal levels by EPI, DOX, or IDA.

A ChIP assay was performed to detect the efficiency of HIF-1α and HIF-2α binding to HREs in the *EPO* (3′ flanking sequence) and *PGK1* (5′ flanking sequence) genes. Figure 3J demonstrates the weak binding of HIF-2α to the *EPO* gene in control cells. The increased binding of HIF-2 to the *EPO* 3′ flanking sequence (induced by CoCl_2_) was inhibited by treatment with EPI, DOX, or IDA. Similarly, all three anthracyclines suppressed the binding of HIF-1 to the 5′ flanking sequence of the *PGK1* gene (Figure 3K). These results indicate that anthracyclines block the binding of HIF-1 and HIF-2 to HREs of target genes, as previously reported [19].

### Anthracyclines suppress metastatic tumor growth and increase survival in a mouse model of metastatic PHEO

**Figure 4:**
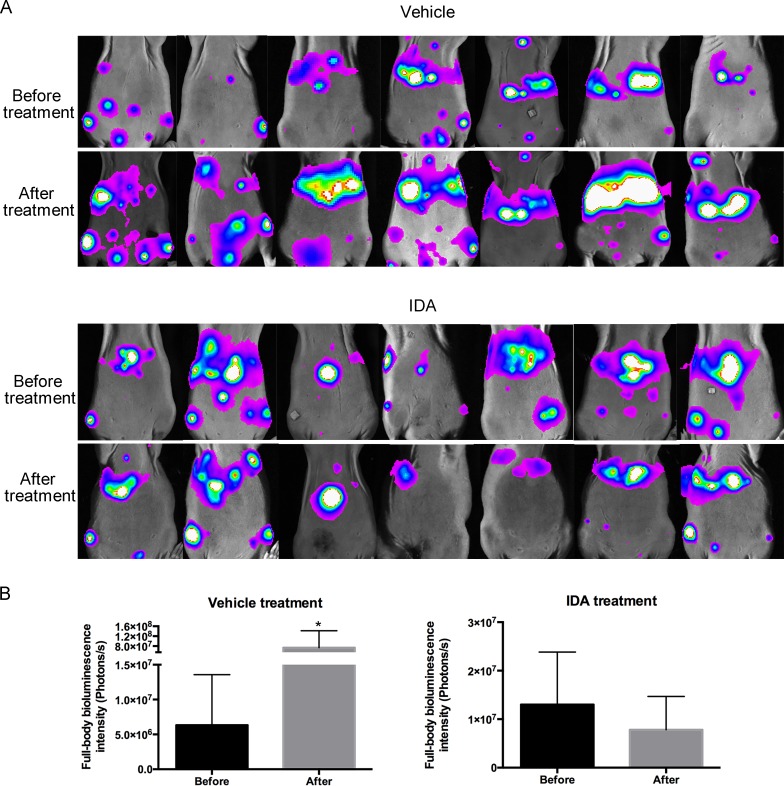
IDA suppressed MTT growth after tail vein injection **A**. Seven mice from each group were subjected to bioluminescence assays before and after treatment. IDA (1 mg/kg per day) was dissolved in 100 μL PBS and injected intraperitoneally for the drug treatment group. Mice from the vehicle group were treated with 100 μL PBS per day. After seven days of treatment, mice were imaged again to measure tumor size. **B**. Quantification of tumors was analyzed based on whole body bioluminescence signal intensity for each group. Before treatment versus after treatment, *P < 0.05.

To assess the effects of anthracyclines on metastatic PHEO *in vivo*, we established a mouse allograft model bearing highly metastatic mouse PHEO cells that constitutively express luciferase (MTT luciferase cells). One million cells were injected into mice through the tail vein. Seven days later, bioluminescence images showed tumor colonization of multiple organs, including the liver and spleen (Figure 4A). Afterwards, IDA treatment began. Following 7 days of treatment with IDA (1 mg/kg/day, intraperitoneal injection), bioluminescence in the vehicle group increased statistically, while mice receiving IDA did not show increased bioluminescence intensity. The differences in full-body bioluminescence intensity before and after treatment in the vehicle and IDA treatment groups are shown in Figure 4B.

**Figure 5:**
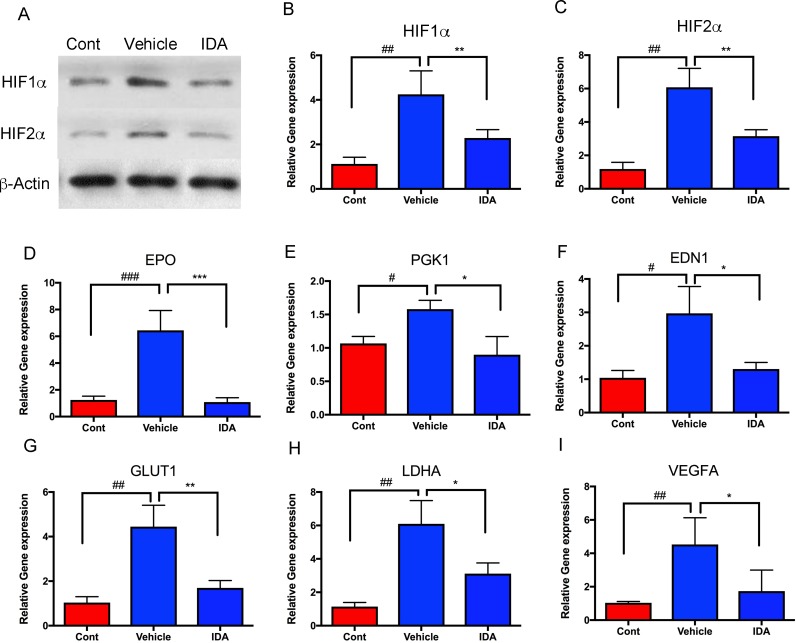
IDA suppressed transcriptional activities of HIFs and their target genes **A**. HIF-1α and HIF-2α protein levels were analyzed in the control (liver without tumor), vehicle (liver with tumors), and IDA treated (liver with tumors) groups. **B**.-**I**. *HIF1A*, *HIF2A*, *EPO, PGK1, EDN1, GLUT1, LDHA,* and *VEGFA* mRNA levels were determined in metastatic tumors in both the vehicle group and IDA treated groups. Normal mouse liver tissue was used as the control. Control versus vehicle, ^#^*P* < 0.05; ^##^*P* < 0.01; ^###^*P* < 0.001. Vehicle versus IDA, *P < 0.05; **P < 0.01; ***P < 0.001.

To analyze the effect of IDA on HIF signaling pathways *in vivo*, tumor allografts were isolated from the livers of the control and treatment groups. Compared to normal livers (control), mRNA and protein levels of HIF-1α and HIF-2α in tumor cells increased in the vehicle group, while the group treated with IDA showed decreased HIF-1α and HIF-2α levels, compared to the vehicle group (Figure 5A, 5B, and 5C). Furthermore, intratumoral expression of HIF target genes (*EPO, PGK1, EDN1, GLUT1, LDHA*, and *VEGFA*) was significantly decreased in tumors after treatment with IDA (Figure 5D-5I). These findings suggest that IDA suppresses metastatic PHEO through inhibition of HIF transcriptional activity.

## DISCUSSION

In the present study, we tested the widely used chemotherapeutic agents, anthracyclines, against experimental PHEO cells under both *in vitro* and *in vivo* conditions. Our findings demonstrate that anthracyclines, especially IDA, suppress PHEO cell proliferation and migration via inhibition of the hypoxia signaling pathway by blocking the DNA binding of HIF-1 and HIF-2. In our experimental animal model, treatment with IDA resulted in a significant reduction in the size of tumors in multiple organs when compared to the control group. Additionally, the hypoxia signaling pathway was significantly suppressed in these tumors. These results suggest that IDA should receive consideration as a promising treatment option for metastatic PHEO/PGL. Based on the significant inhibition of HIF DNA binding and downstream target gene expression, this treatment option may be particularly effective in tumors with *HIF2A* mutations [16, 19], as well as in those with mutations in other PHEO/PGL susceptibility genes that cause upregulation of the hypoxia signaling pathway, such as *EGLN1* and *VHL* [24, 25].

Anthracyclines have been used as chemotherapeutic agents for many years, and the anti-cancer effect is mainly attributed to intercalating DNA, stabilizing the topoisomerase II complex, and further preventing DNA replication [26, 27]. Apart from their major use in the treatment of acute myeloid leukemia and acute lymphocytic leukemia, anthracyclines are also effective in treating neuroblastoma [28], another adrenal neural crest tumor producing catecholamines and their metabolites, which is also seen in PHEO/PGL. Previous anecdotal evidence reported successful results in a patient with metastatic PHEO treated with a combination of an anthracycline and the conventional CVD regimen (so called ACVD) [23]. After ACVD treatment termination, the patient remained in complete remission, with no recurrence, for three years. Anthracyclines have also been successfully used in selective arterial chemoembolization in patients with hepatic metastases from PHEO and medullary thyroid carcinoma [29, 30]. However, the use of anthracyclines as chemotherapeutic agents in the treatment of metastatic PHEO/PGL has not been systematically evaluated.

HIF-1α and HIF-2α are the two best-studied isoforms of HIFs, and are now considered important therapy targets for various tumor types [31-33]. HIF-1α and HIF-2α are commonly upregulated during cancer progression [34, 35]. This is also the case in PHEO/PGL, especially those that belong to cluster 1, where the role of the hypoxia signaling pathway has been previously established [16, 25, 36, 37]. Moreover, it has recently been proposed that mutations in PHEO/PGL susceptibility genes in cluster 2 can also converge on the hypoxia signaling pathway, resulting in its upregulation [14]. Finally, novel somatic and germline mutations of *HIF2A*, which result in a syndrome consisting of multiple and recurrent PHEOs/PGLs, duodenal somatostatinoma, and polycythemia, have recently been discovered [16, 38, 39].

In the present study, we demonstrated the effects of three different anthracyclines on tumor growth suppression by blocking DNA binding of HIF-1 and HIF-2 resulting in reduced transcription of hypoxia response genes. The expression of these HIF-1 and HIF-2 target genes involved in angiogenesis, tumor metastasis, and polycythemia, was significantly decreased in both cell lines and tumors after treatment with anthracyclines. Other groups have reported the inhibitory effect of DOX on HIF-1 [40] and its downstream target genes [19], which further supports our findings.

IDA, without the 4-methoxy group, is an analog of DAU, and has a broader spectrum as a chemotherapy agent compared to the other anthracyclines [41]. Historically, IDA has been combined with cytosine arabinoside as a first line therapy for acute myeloid leukemia (AML). Our results demonstrate the efficiency of IDA in not only blocking DNA binding like other anthracyclines, but also in the reduction of HIF-1α and HIF-2α protein levels. Based on previous findings, IDA, or IDA in combination with triptolide, also suppresses the expression of HIF-1α and related downstream target genes in leukemia stem cells [42]. Shaul *et al.* [43] showed that IDA decreases cell viability significantly faster and stronger than DOX, which is likewise consistent with our results. Their group suspected different underlying chemotherapeutic mechanisms for IDA and DOX, and suggested that IDA has additional undiscovered cellular targets compared to DOX. From our data, IDA specifically causes downregulation of HIF-1α and HIF-2α which was not observed with the other two anthracyclines in this study. IDA has also been shown to block binding of HIF-1 to HREs in HIF target genes [19].

Anthracyclines, like other chemotherapeutic agents, cause cytotoxicity of normal proliferating cells, leading to side effects such as weight loss, headaches, mucositis, increased skin pigmentation, nausea, and myelosuppression [44, 45]. Additionally, they are associated with acute and chronic dose limiting cardiotoxicity (with IDA being the least cardiotoxic), which can severely impair cardiac function, resulting in heart failure [46]. Similar findings were previously reported when a patient with metastatic PHEO receiving ACVD chemotherapy developed grade I bone marrow suppression and cardiovascular instability [23]. Therefore, an individualized approach is necessary when considering this regimen, either alone or in synergistic combinations with other agents, especially in pediatric patients and adults with multiple comorbidities [47].

Our results indicate that anthracyclines, especially IDA, significantly inhibit *in vitro* cell growth and *in vivo* tumor formation in a mouse model of PHEO. Apart from the well-known, multiple, chemotherapeutic mechanisms of anthracyclines, we establish anthracycline-induced suppression of the hypoxia signaling pathway. This occurs through inhibition of HIF-1 and HIF-2 binding to the HRE sites on DNA, resulting in decreased transcription of downstream target genes involved in apoptosis, migration, angiogenesis, and metastasis. Our results suggest that chemotherapy with anthracyclines, either alone or as adjuvant therapy, should be considered as a treatment option in patients with metastatic PHEO/PGL, especially those harboring mutations in the *HIF2A* gene or other PHEO/PGL susceptibility genes that cause upregulation of the hypoxia signaling pathway.

## MATERIALS AND METHODS

### Cell culture and drug treatment

Hep3B cell lines were purchased from the American Type Culture Collection (ATCC). Mouse tumor tissue (MTT) cells were derived from a liver metastasis in a mouse pheochromocytoma cell (MPC) allograft model, as previously described [48, 49]. Cells were cultured in DMEM with 10% FBS (Gemini Bioproducts) at 37°C, with 5% CO_2_ (v/v). Cells were treated with 100 μM CoCl_2_ (Sigma-Aldrich) for 16 hours. EPI, DOX, and IDA (Sigma-Aldrich) were used to treat cultures.

Hep3B-HRE-luciferase and MTT-luciferase subclones were established by transfection with Cignal Lenti HIF Reporter (Luc) lentivirus (Qiagen) and pLLuc retroviral vector, respectively. Stable cell lines expressing luciferase were selected by 5 μg/mL puromycin (Sigma-Aldrich) or 1000 μg/mL G418 (Life Technologies). A luciferase reporter assay was performed according to manufacturer protocol using the ONE-Glo Luciferase Assay System (Promega). Cycloheximide (CHX) was purchased from Sigma and cells were treated on different time courses (0, 15, 30, and 60 min) at 100 μM. Small interfering RNA (siRNA) targeting HIF-1α and HIF-2α (Abnova) were transfected into Hep3B cells for 48 hours using Lipofectamine RNAiMAX reagent (Invitrogen).

### Cell proliferation assay

Cell proliferation was measured by the Cell Counting Kit-8 (CCK8, Dojindo, Japan). MTT or Hep3B cells were seeded in a 96-well plate with 2×10^4^ cells/well. The number of surviving cells was assessed following administration of varying treatment protocols.

### Cell migration assay

Hep3B cells were seeded in a 35-mm μ-Dish with 2-well culture-insert (iBidi, USA) at a density of 3×10^5^. The culture dish was incubated for 24 hours and the culture-insert was removed. Cells were washed with fresh growth media and incubated in the presence of a candidate compound. Cell morphology was recorded with a Nikon BioStation IM-Q for 24 hours, and imaging was performed every 5 minutes.

### Reverse-transcription (RT) and quantitative PCR (qPCR)

Total RNA from tissue or cell cultures was extracted using the NucleoSpin RNA Purification Kit (Machery-Nagel). DNase was used to digest genomic DNA according to the manufacturer's instructions. One microgram of RNA was used for reverse transcription using the Super Script III First-Strand Synthesis SuperMix (Invitrogen). Target genes were amplified using the ViiA 7 real-time PCR system (Applied Biosystems). Primers used in qPCR were as follows: *HIF1A* (forward: ACC TGA GGA GAG GCT CGG; reverse: ACT TAT CTT TTT CTT GTC GTT CGC); *HIF2A* (forward: GCG CAC CTC GGA CCT TCA; reverse: TCT CCG AGC TAC TCC TTT TCT TC); *EPO* (forward: GCT GCA TGT GGA TAA AGC CCG; reverse: CAC ACC TGG TCA TCT GTC CC); *β-actin* (forward: TCA GAA GGA TTC CTA TGT GGG CGA CGA; reverse: TCC CAG TTG GTG ACG ATG CCG T); *PGK1* (Qiagen QT), *GLUT1* (Origene HP209446), *VEGFA* (Qiagen QT01682072).

### Western blot analysis

For total cellular protein concentration, cells were washed with PBS three times and lysed with RIPA lysis buffer with a complete protease inhibitor cocktail (Roche, Sweden). Whole cells were lysed for 20 minutes on ice and centrifuged for 20 minutes at 4°C. A Bio-Rad protein assay kit was used to measure protein content in the supernatant, and equal amounts of samples were electrophoresed in NuPAGE Bis-Tris 4-12% gel (Invitrogen, USA). Proteins were transferred onto PVDF membranes (Millipore), and blocked with 5% nonfat milk. Target proteins were probed with primary antibodies of HIF-1α (Sigma), HIF-2α (Abcam), and β-actin (Cell Signaling Technology) at 4°C overnight, and then incubated in HRP-conjugated secondary antibodies at room temperature for 1 hour. Signals were detected with an enhanced chemiluminescence ECL kit (Pierce).

### Chromatin immunoprecipitation (ChIP) assay

Cell cultures were treated with different anthracyclines, and 37% formaldehyde was added to crosslink protein-DNA complexes. ChIP analysis of the HIF-1α (Sigma) or HIF-2α (Abcam) binding to HRE sites of *PGK1* or *EPO* was performed according to manufacturer protocol using the MAGnify Chromatin Immunoprecipitation System (Life Technologies). The binding activities between HIF-1α or HIF-2α and target genes were measured by PCR with ChIP primers for *EPO* (forward: TCC AAA TCC CCT GGC TCT GT; reverse: ATT GAC CAG CGT AGG CAG AG) and *PGK1* (forward: CAT TCT TCA CGT CCG TTC GC; reverse: TCT GCG AGG GTA CTA GTG AGA). Samples were run for 25 cycles (95°C for 20 seconds, 57°C for 20 seconds, and 68°C for 30 seconds). PCR products were separated on 1% agarose gel with SYBR Safe staining (Invitrogen).

### Allograft assay and *in vivo* imaging

Animal experiments were approved by the *Eunice Kennedy Shriver* NICHD animal protocol (ASP: 15-028). Six week-old female nude athymic mice (nu/nu) were purchased from The Jackson Laboratory. The weight of each mouse was approximately 20 g at experiment onset. Mice were injected into the tail vein with 1 million MTT luciferase cells in 100 μL PBS. Animals were imaged from day 7 post tail-vein injection by bioluminescence imaging. Mice injected with MTT luciferase allografts received an I.P. injection of 10 μL/g of body weight of D-luciferin (Caliper Life Sciences) dissolved in 100 μL PBS. The *in vivo* imaging system (Bruker) and Bruker MI SE Software were utilized to acquire and analyze the signaling. Seven days after tail vein injection, all mice were randomized into two groups, each consisting of 7 mice. Mice were either treated with solvent alone, or IDA hydrochloride intraperitoneal injection (Pfizer) with a 1 mg/kg dose every day, for seven days. Animals were re-imaged to measure the size of metastatic tumors. Euthanasia by cervical dislocation followed. Liver and spleen tumors were removed. Expression profiles of HIF-1α and HIF-2α downstream target genes were measured using qPCR according to the protocol described above.

### Statistical analysis

Data was analyzed by one-way factorial ANOVA combined with Tukey's multiple-comparisons test or Student's *t*-test using Prism 7.0 statistical software (Graph Pad Software). A *P* value of < 0.05 was considered statistically significant. Graphic representations were expressed as mean ± SEM.

## Abbreviations

PHEOs: Pheochromocytomas
PGLs: paragangliomas
DAU: daunorubicin
DOX: doxorubicin
EPI: epirubicin
IDA: idarubicin
HRE: hypoxia response element
EPO: erythropoietin
PGK1: phosphoglycerate kinase 1
EDN1: endothelin 1
GLUT1: glucose transporter 1
LDHA: lactate dehydrogenase A
VEGFA: vascular endothelial growth factor
HIFs: hypoxia-inducible factors
MTT: mouse tumor tissue
IC_50_: half maximal inhibitory concentration
AML: acute myeloid leukemia
CHX: cycloheximide
siRNA: small interfering RNA.
